# Sequence-specific radiomics for diagnosis of spinal bone loss

**DOI:** 10.3389/fendo.2026.1823826

**Published:** 2026-04-29

**Authors:** Tingyu Xue, Yaguang Li, Huayi Zhao, Tianzi Zhang, Jiayi Wang, WenHao Jiang, Licun Lei, Yong Wang

**Affiliations:** 1 Department of Radiology and Nuclear Medicine, The First Hospital of Hebei Medical University, Hebei, China; 2 School of Medical Imaging, Hebei Medical University, Hebei, China

**Keywords:** abnormal bone density, lumbar vertebra, machine learning, magnetic resonance imaging, osteoporosis, radiomics

## Abstract

**Objective:**

To establish a sequence-specific predictive model for spinal bone loss by leveraging conventional lumbar MRI, targeting abnormal bone density or osteoporosis differentiations.

**Methods:**

A total of 320 MR scans from 160 patients (52 men and 108 women; mean age 61.27 ± 12.72 years) who underwent lumbar MRI and quantitative computed tomography (QCT) examinations were retrospectively enrolled in this study cohort. Radiomic features were extracted from the lumbar spine MR images. With QCT as the reference standard, six radiomic-based machine learning models including K-nearest neighbor (KNN), support vector machine (SVM), Linear Discriminant Analysis (LDA), logistic regression (LR), stochastic gradient descent (SGD), Gaussian NB were developed to predict abnormal bone density and osteoporosis using T1WI alone, T2WI alone, and the combined T1WI+T2WI. The dataset was randomly split into a training/validation set and a testing set in a 7:3 ratio. The performance metrics of the models were calculated and evaluated.

**Results:**

Among the six machine learning models evaluated, T1WI and T2WI each exhibited prominent advantages for predicting osteoporosis and abnormal bone mass, respectively. Take KNN as an example. T1WI achieved the highest AUC (0.821) for predicting osteoporosis on test set (mean of 10 repeated evaluations), significantly higher than T2WI (AUC = 0.782) and the combined T1WI+T2WI approach (AUC = 0.775). In contrast, T2WI demonstrated superior performance for the prediction of abnormal bone density, with an AUC of 0.942 (T1WI and T1WI+T2WI were 0.884 and 0.923, respectively).

**Conclusion:**

Our investigation into predicting abnormal bone density and osteoporosis from lumbar spine MRI sequences shows that predictive efficacy is sequence-dependent. T1WI features proved more effective for osteoporosis identification, while T2WI features were better for abnormal bone density prediction, highlighting the importance of sequence selection based on target pathology.

## 1 Introduction

Osteoporosis, a systemic skeletal degenerative disorder, manifests through progressive deterioration of bone microstructure and reduced bone mineral density (BMD), culminating in heightened fracture susceptibility (1, 2). This condition disproportionately affects aging populations, and its global prevalence is projected to escalate alongside demographic aging trends. Epidemiological models predict that China will face approximately 5.99 million osteoporotic fracture cases by 2050 (3, 4). Early identification of at-risk individuals remains a critical priority in clinical management (5).

Current diagnostic protocols predominantly rely on radiological bone mineral density (BMD) assessments. While dual-energy X-ray absorptiometry (DXA) remains the gold standard (6, 7), its accuracy for lumbar spine evaluations is frequently compromised by anatomical confounders such as facet joint degeneration, vertebral endplate deformities, and vascular calcifications (8, 9). Quantitative computed tomography (QCT) overcomes these limitations through volumetric BMD quantification (10). However, this enhanced precision is achieved at the cost of substantially higher ionizing radiation exposure compared to DXA (11).

In fact, being one of the most common imaging approaches without ionizing radiation, MRI is extensively utilized in the assessment of lumbar disorders (11–13). However, technical challenges persist in visualizing osseous tissues due to the inherent low proton density and rapid signal decay in mineralized matrices. Recent investigations have identified bone marrow fat fraction (BMFF) as a promising biomarker for osteoporosis severity (9). Chemical shift encoding-based water-fat separation sequences allow for providing accurate BMFF by means of single-voxel magnetic resonance spectroscopy (MRS) (14, 15). However, there is an absence of large-sample studies further confirms the diagnostic performance (16). In addition, obesity, inflammation or metabolic diseases (such as diabetes) may interfere with the accuracy of fat quantification (17). Therefore, in addition to the simple BMFF average, the discovery of more quantitative features remains to be explored.

The integration of artificial intelligence (AI) in medical imaging has enabled transformative advances by identifying distinct texture and shape characteristics within osseous lesions that are otherwise difficult to quantify visually (18, 19). Notably, AI-based radiomics approaches have shown encouraging performance in other orthopedic conditions such as osteonecrosis, osteoarthritis, and bone tumors (20, 21). Likewise, AI has seen extensive application in the prediction of osteoporosis. Kang et al. validated machine learning classifiers for osteoporosis detection via T2WI (22), while Zhao et al. established a radiomics using short-axis mDixon MRI to predict osteoporotic risk (23). Complementary work by F. G. et al. developed machine learning methods to estimate bone mineral density and detect abnormal bone density/osteoporosis from conventional T1WI/T2WI and planar radiography (24). All the aforementioned studies have yielded satisfactory and commendable outcomes (18). However, no systematic comparison exists regarding the diagnostic performance of machine learning models utilizing isolated T1WI, T2WI, or their synergistic combination.

**Scheme 1:**
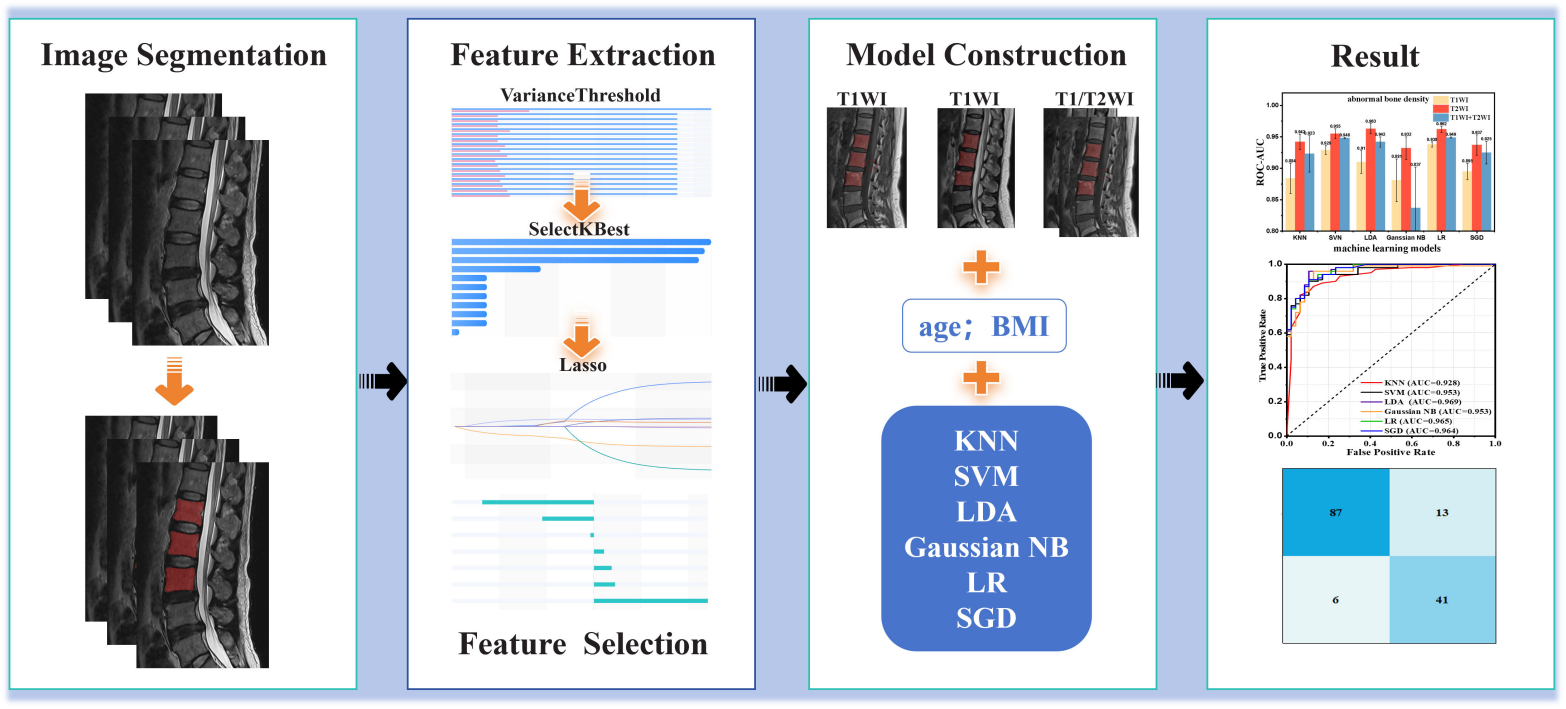
Schematic illustration of the architecture for classification model, includes image segmentation, feature extraction and selection, model construction and result.

In the present study, we introduce radiomics technology to establish an osteoporosis/abnormal bone density prediction model based on T1WI and T2WI image features (**Scheme 1**). We find that T1WI and T2WI showed the different performance for predicting osteoporosis/abnormal bone density. This indicates that the choice of MRI sequence significantly impacts prediction accuracy and should be tailored to the specific bone condition being assessed. The development of MRI-based strategies holds significant potential in diagnosis of osteoporosis, while avoiding radiation exposure.

## 2 Materials and methods

### 2.1 Study participants

A retrospective analysis was conducted on patients who underwent lumbar MRI and QCT examinations at the First Hospital of Hebei Medical University between 2022 and June 2025. After screening and evaluation, 26 of 186 candidate patients were excluded. The remaining 160 patients meeting the inclusion criteria were enrolled in the study: (1) adults aged ≥20 years; (2) Completion of simultaneous lumbar spine MRI and QCT examinations within three months. Exclusion criteria: (1) Presence of lumbar metal implants, images with metal artifacts or poor quality affecting analysis; (2) History of abnormal bone metabolism from any cause or diseases such as multiple myeloma. The clinical information of the patients, including age, gender, body mass index (BMI) and BMD were collected. All patients divided into the osteoporosis/non-osteoporosis group or abnormal bone density/non-abnormal bone density group according to the QCT results: BMD > 120 mg/cm^3^ indicates normal bone mineral density, 80–120 mg/cm^3^ is considered low bone mass, and BMD < 80 mg/cm³is osteoporosis. BMD refers to the average value of the vertebral body BMD of L1-L3. This retrospective investigation obtained permission from the ethics committee our hospital, with no requirement of informed consent from patients.

### 2.2 Image acquisition

MRI were performed using a Siemens Prisma 3.0T scanner. The scanning parameters are following: T1WI: TR 400 ms, TE 8.6 ms, FOV 280 × 280 mm, matrix 320 × 256, flip angle 130°, slice thickness 4 mm, scan time 38 s; T2WI: TR 3200 ms, TE 95 ms, FOV 280 × 280 mm, matrix 320 × 226, flip angle 160°, slice thickness 4 mm, scan time 125s.

CT examinations were performed using a Philips Incisive 64-slice spiral CT scanner (tube voltage: 120 kV, tube current: 150 mAs, FOV: 150 mm × 150 mm). The scanning range extended from L1 to the L5. BMD (L1-L3) was measured on Mindways QCT Pro system(Mindways Software Inc., Austin, TX, USA): The QCT Pro software automatically segmented rectangular Regions of interest (ROIs) on CT sagittal images of L1-L3 vertebrae and elliptical ROIs on axial images at the vertebral body center. ROIs were verified and refined by radiologists with >3 years of experience for accuracy. Volumetric BMD within ROIs was quantified (unit: mg/cm³ of calcium hydroxyapatite), and the final BMD was the mean BMD of L1-L3 vertebral bodies.

All the images were derived from the picture archiving and communication system (PACS) of the Department of Radiology and Nuclear Medicine of the First Hospital of Hebei Medical University and downloaded in DICOM format.

### 2.3 Radiomics

#### 2.3.1 Image segmentation

**Figure 1:**
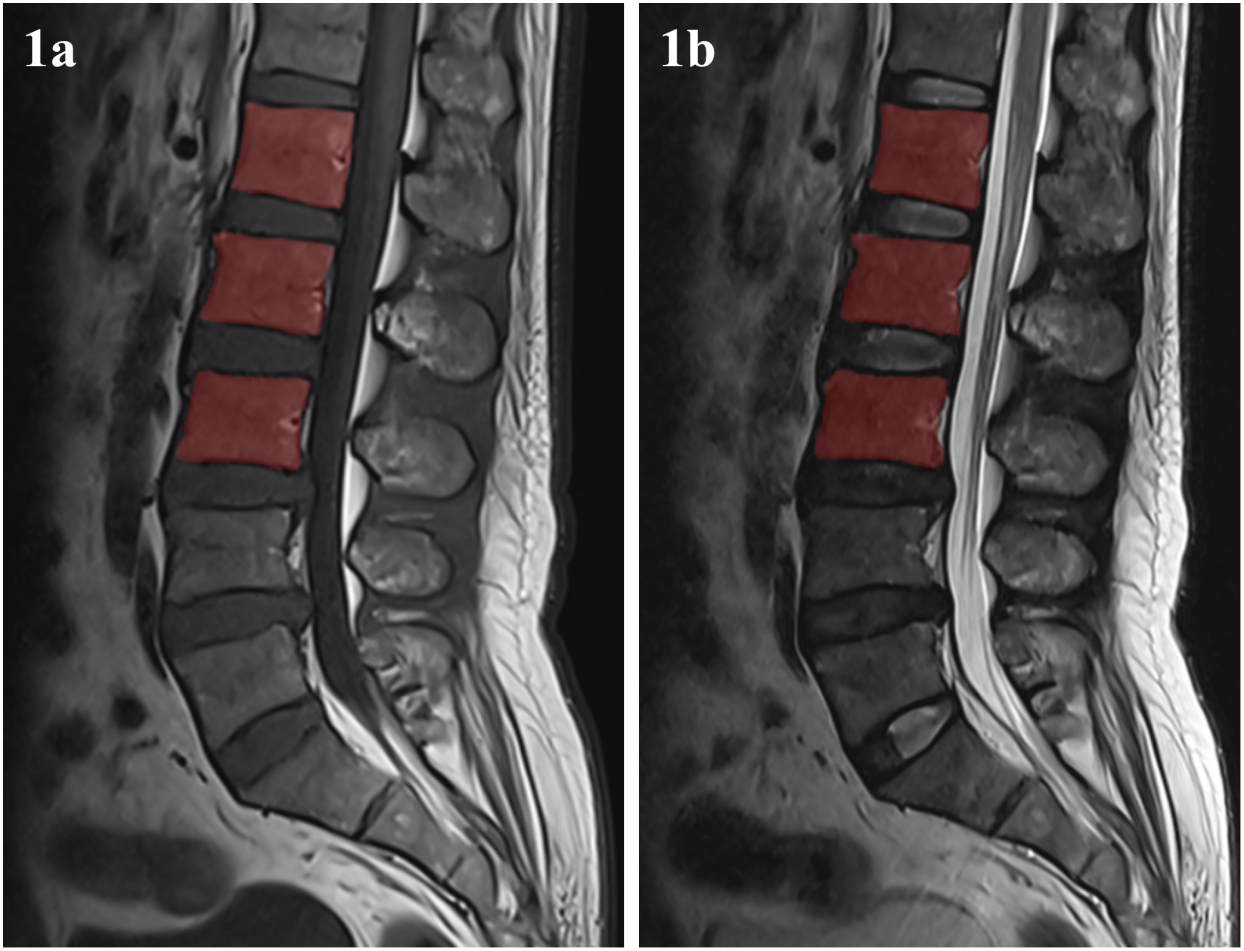
Representative volume-of-interest (VOI) in MR image of the lumbar spine. **(a)** and **(b)** are respectively the sagittal images of T1WI and T2WI.

The sagittal T1WI and T2WI lumbar spine images were imported into the Digital Intelligence Precision Surgery System (Shenzhen Xudong Digital Medical Imaging Technology Co., Ltd., http://ai.yorktal.com/login). ROIs corresponding to the L1–L3 vertebral bodies were manually delineated slice-by-slice on the 2D sagittal image series; the sequentially segmented 2D ROIs were then stacked to construct a complete three-dimensional volume-of-interest (VOI) covering the entire L1–L3 vertebrae. An attending radiologist (3 years of experience in musculoskeletal imaging) performed manual refinement of the complete vertebral contour, excluding vertebral endplates, paravertebral soft tissues, and adjacent intervertebral discs (**Figure 1**). All refined contours underwent independent review by a senior radiologist (7 years of subspecialty experience). In cases of inter-observer discrepancy, consensus was established through consultation with a third radiologist (>15 years of experience).

#### 2.3.2 Radiomics features

Digital Intelligence Precision Surgery System was used to extract radiomic features. It includes seven common feature groups: first order features; shape features; Gray-level dependence matrix; Gray-level co-occurrence matrix; Gray-level run length matrix; Gray-level size zone matrix; neighboring gray tone difference matrix. A total of 1688 quantitative imaging features were extracted from MR images.

To reduce feature dimensionality, we implemented a three-stage feature selection pipeline. First, features exhibiting low variance were removed using VarianceThreshold with a threshold of 0.8. Next, features significantly correlated with the classification outcome were selected via SelectKBest, retaining those with a p < 0.05. Finally, to further refine the feature set and mitigate overfitting, we applied the Least Absolute Shrinkage and Selection Operator (Lasso). Lasso employs L1 regularization to shrink the coefficients of weakly relevant or irrelevant features towards zero. Features with non-zero Lasso coefficients were selected for the regression model and subsequently incorporated into the final radiomics feature set.

#### 2.3.3 Prediction model development and validation

Three predictive models were developed (**Scheme 1**): T1WI model, T2WI model, T1WI+T2WI model. T1WI model: features were extracted solely from the T1WI sequences with clinical features to construct the model. T2WI model: features were extracted solely from the T2WI sequences with clinical features to construct the model. T1WI+T2WI model: features were extracted concurrently from both T1WI and T2WI sequences with clinical features to construct the combined model. Using the selected features and clinical characteristic, two classification model was constructed to predict abnormal bone density or osteoporosis. In addition, a total of six machine learning algorithms were used in this study: K-nearest neighbor (KNN), support vector machine (SVM), Linear Discriminant Analysis (LDA), logistic regression (LR), stochastic gradient descent (SGD), Gaussian NB.

To train and evaluate the performance of the above models, the available data were randomly split into a set used for training and cross-validation (70%) and a set used for testing the performance of model (30%). 10-fold cross-validation was nested within the training set to conduct model training, three-stage radiomic feature selection, parameter tuning. After obtaining the optimized model parameters and feature sets via 10-fold cross-validation on the training set, the mean values of performance metrics from 10 repeated evaluations on the independent test set were reported as the final model performance. Model performance was evaluated using metrics including the area under the receiver operating characteristic curve (AUC), accuracy, specificity, sensitivity and F1-score. Elevated AUC signifies enhanced model discriminability. (Mean performance metrics from 10 repeated evaluations on the test set are reported in the Abstract and **Supplementary Material**, with a single representative run presented in the Result text for visual comparison).

### 2.4 Statistical analysis

Statistical analyses were conducted using IBM SPSS Statistics (Version 27; Armonk, NY). Continuous variables underwent normality assessment via Shapiro-Wilk tests and homogeneity of variance evaluation with Levene’s test. Normally distributed data (defined as p > 0.05 on both tests) were compared using independent samples t-tests, with results presented as mean ± standard deviation (X ± S). Non-normally distributed variables were analyzed with Mann-Whitney U tests, reported as median with interquartile range (IQR). Categorical variables were assessed via Pearson’s chi-square tests or Fisher’s exact tests (for cell counts < 5), expressed as frequency counts and percentages.

## 3 Result

A total of 160 patients were enrolled in this study, comprising 52 males (31.25%) and 108 females (68.75%), with an age range of 20 to 86 years and BMI of 11 to 50.8 kg/m^2^. Among the three clinical indicators (age, sex, and BMI), only age and BMI showed statistically significant differences between osteoporosis (n = 89) and non-osteoporosis (n = 71) groups on the entire dataset (p < 0.05). No significant differences were observed for sex (p > 0.05; **Table 1**). Similar results were found for abnormal (n = 126) vs. non-abnormal bone density (n = 34) classifications.

**Table 1:** Univariable analysis of clinical data.

Classification tasks	Variables	OR	95%CI	z	p-value
Osteoporosis/non-osteoporosis	age	0.9614	0.9560-0.9669	-13.6186	0
	sex	1.0442	0.8662-1.2588	0.4537	0.65
	BMI	1.0423	1.0211-1.0638	3.9612	0.0001
abnormal bone density/normal bone density	age	1.0232	1.0162-1.0302	6.5803	0
	sex	1.1082	0.9420-1.3038	1.2393	0.2152
	BMI*	0.9667	0.9493-0.9844	-3.6545	0.0003

*BMI Body Mass Index.

**Figure 2:**
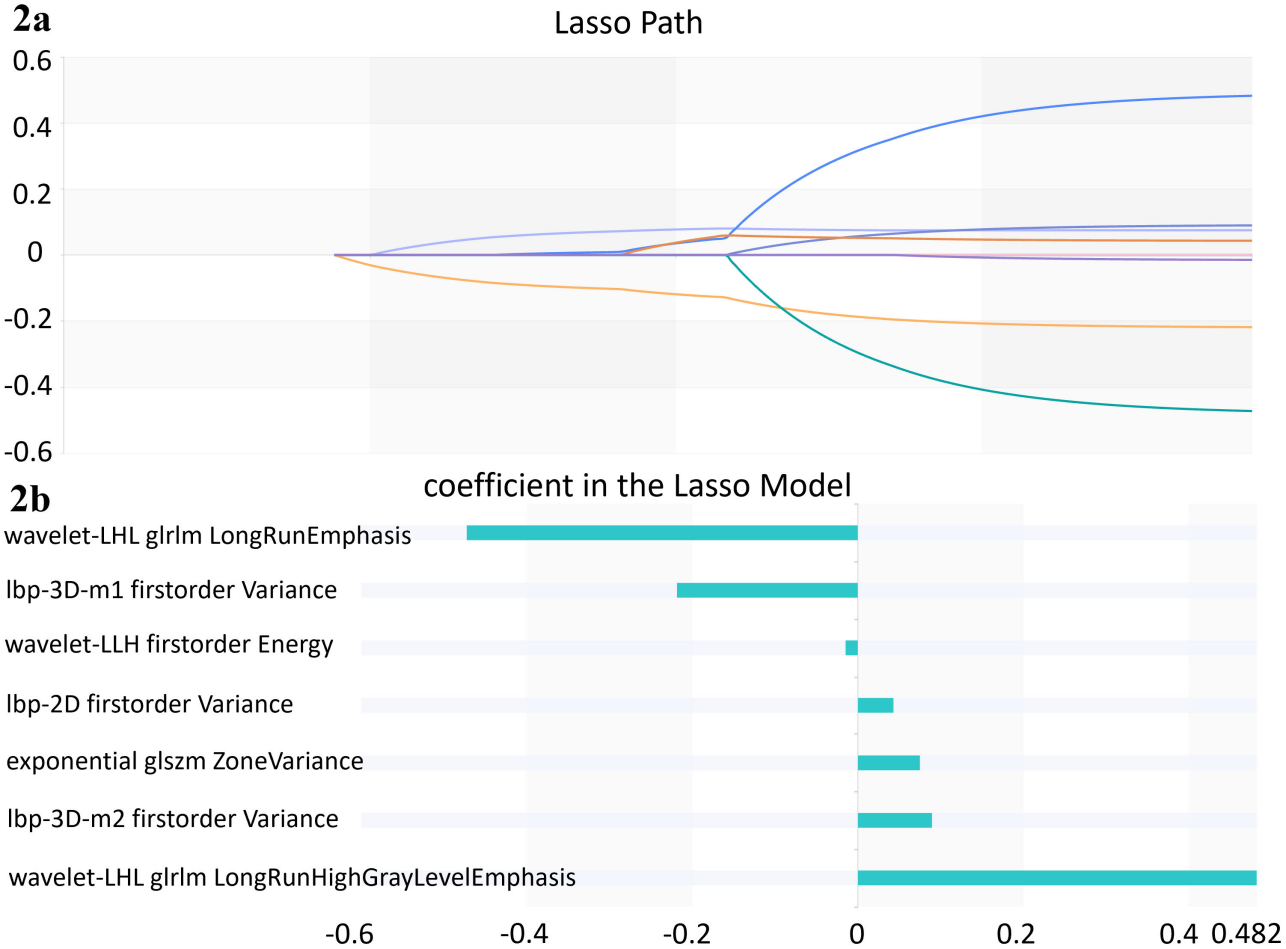
One of the Lasso algorithms in 10-fold cross-validation classification model for osteoporosis with T1WI. **(A)** Lasso path. **(B)** coefficients in Lasso model, 7 features which are correspond to the optimal alpha value were selected.

During 10-fold cross-validation, multiple radiomics features were obtained. For example, concerning the detection of osteoporosis, 1,688 quantitative radiomics features were extracted from T1WI for osteoporosis detection. These features comprised 16 categories: wavelet-HHH, wavelet-LLH, wavelet-LHL, wavelet-LHH, wavelet-HLL, wavelet-HLH, wavelet-HHL, wavelet-LLL, exponential, logarithm, square, square root, gradient, lbp-2D, lbp-3D-m1, and lbp-3D-k. To reduce redundancy, feature selection was performed in three stages: Variance thresholding identified 383 non-redundant features (**Supplementary Figure 1A**); SelectKBest retained 12 discriminative features (**Supplementary Figure 1B**); LASSO regression finalized 7 optimal features (**Figure 2**). A synthesis of all selected radiomic features revealed that core features for osteoporosis prediction with T1WI were dominated by Variance-related metrics, including lbp-3D-m1_firstorder_Variance and exponential_glszm_ZoneVariance. In contrast, the core features identified from T2WI for abnormal bone density prediction comprised Kurtosis and NonUniformity metrics, namely exponential_firstorder_Kurtosis, wavelet-LLL_glszm_SizeZoneNonUniformity and exponential_glszm_GrayLevelNonUniformity. These core features were completely non-overlapping between the two sequences, which exhibited a distinct sequence-specific distribution pattern (**Supplementary Tables 3**, **4**).

**Figure 3:**
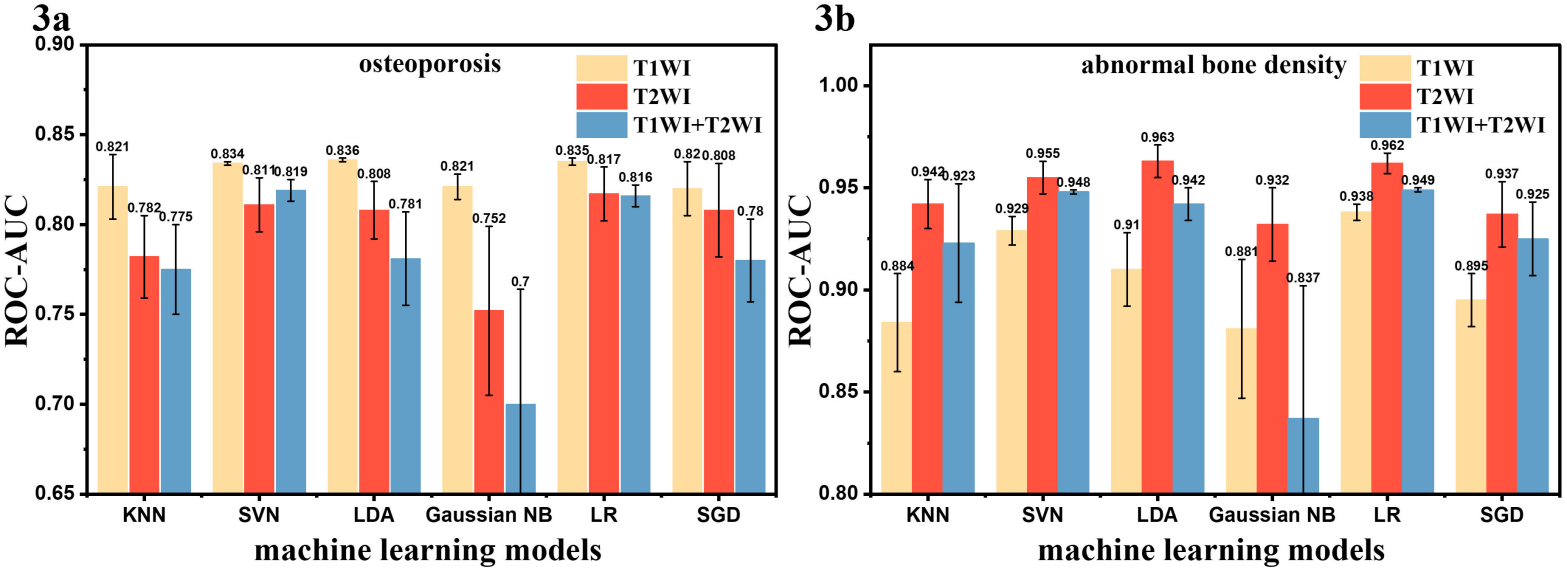
Mean AUC for osteoporosis **(A)** or abnormal bone density **(B)** prediction on test set (10 repeated evaluations).

**Figure 4:**
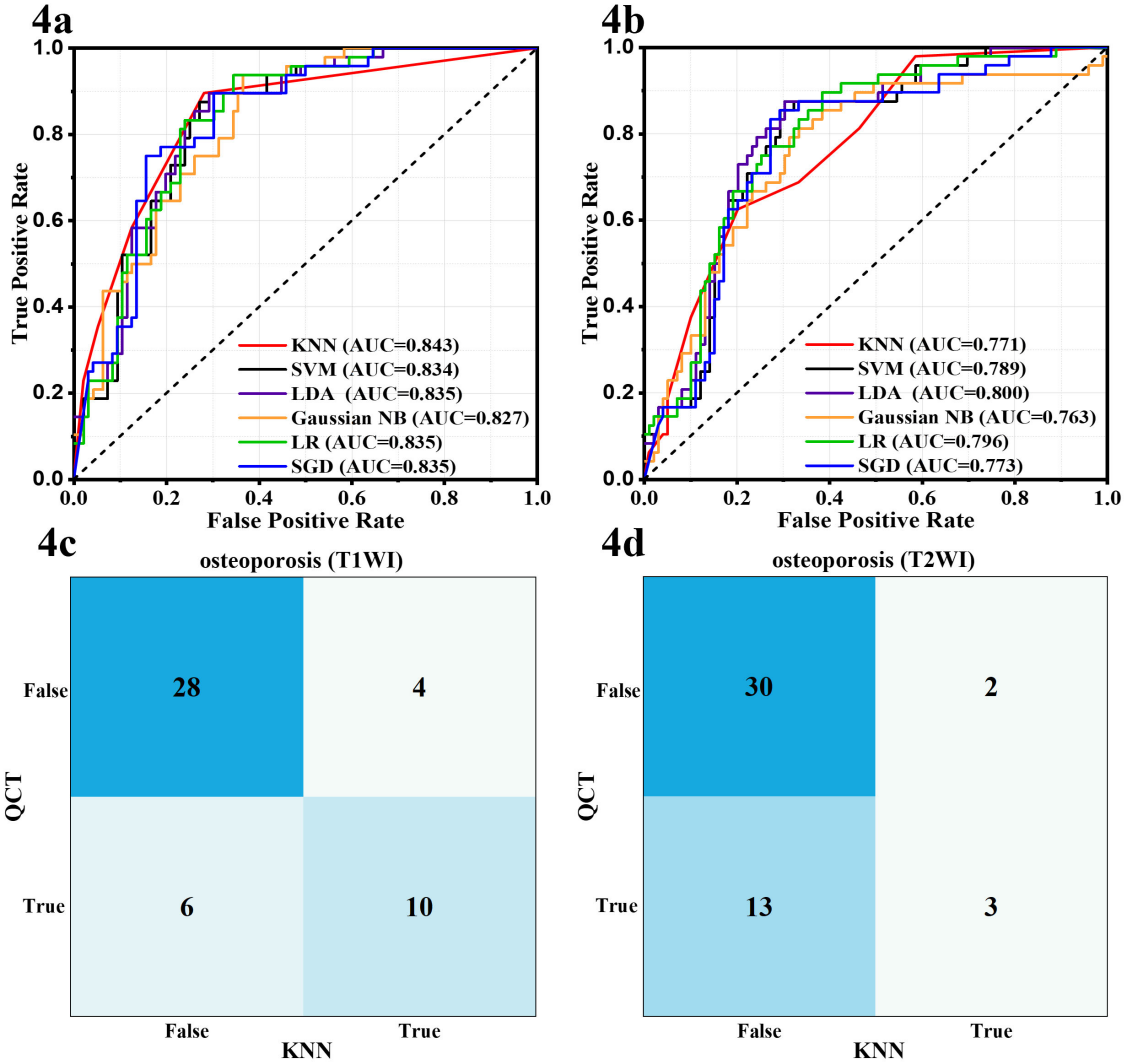
Receiver operating characteristic curves and confusion matrices of the six machine learning models for the prediction of osteoporosis based on clinical data and T1WI **(A, C)** or T2WI **(B, D)**.

**Figure 5:**
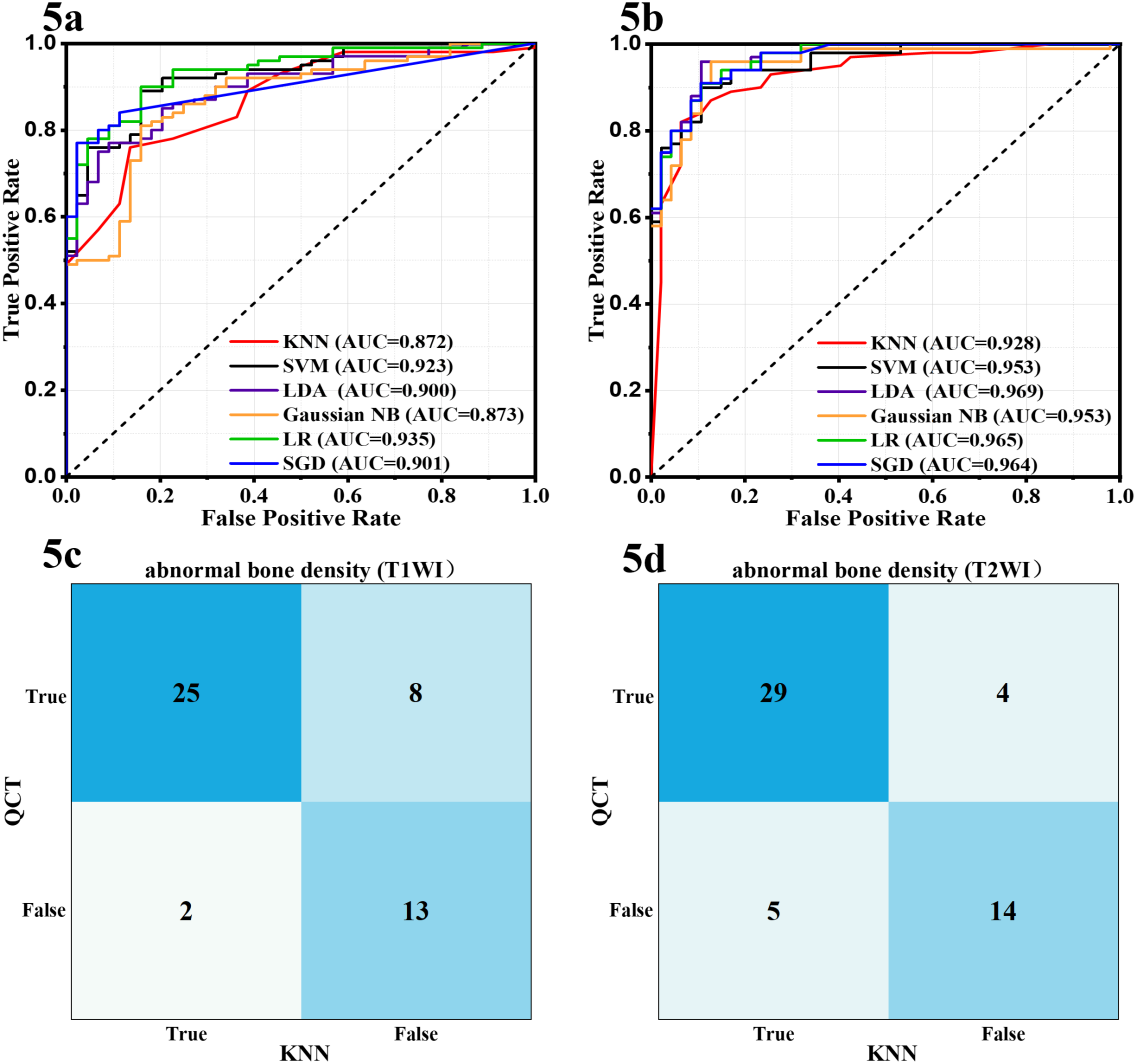
Receiver operating characteristic curves and confusion matrices of the six machine learning models for the prediction of abnormal bone density based on clinical data and T1WI **(A, C)** or T2WI **(B, D)**.

Based on the selected radiomic features and clinical characteristics, two classification models were constructed. As shown in **Supplementary Tables 1, 2**, the T1WI and T2WI groups showed different superiority in two classification model. In the prediction of osteoporosis, T1WI-based models significantly outperformed T2WI and combined T1WI+T2WI approaches across all machine learning algorithms. The AUC-ROC values and accuracy of all machine models were higher than those of the T2WI groups considering the mean value of the 10 repeated evaluations on the test set (**Figure 3A**, **Table 2**, **Supplementary Table 1**), with KNN being prominent for models (**Figure 4**, **Supplementary Figure 2**, AUC-ROC: 0.843 vs 0.771 vs 0.766, accuracy: 0.762 vs 0.707 vs 0.735 for T1WI, T2WI and T1WI+T2WI, respectively). However, in the prediction of abnormal bone density, the opposite results were observed. T2WI demonstrated consistently superior performance across all machine learning models (**Figure 3B**, **Table 2**), exceeding T1WI and T1WI+ T2WI in sensitivity, specificity, accuracy (**Supplementary Table 2**). Critically, this advantage peaked in the KNN framework, where T2WI achieved 0.964 AUC and accuracy of 0.871, surpassing T1WI-KNN (0.895/776) and T1WI+T2WI-KNN (0.909/0.787) (**Figure 5**, **Supplementary Figure 3**). Furthermore, it is worth noting that the combined T1WI+T2WI approach underperformed single-sequence models in both classification tasks.

**Table 2:** Mean accuracy for two classification models on test set (10 repeated evaluations).

Classification	Group	KNN	SVM	LDA	LR	SGD	GaussianNB
osteoporosis	T1WI	0.761	0.768	0.771	0.761	0.761	0.733
	T2WI	0.724	0.744	0.733	0.759	0.721	0.715
	T1WI+T2WI	0.748	0.745	0.748	0.751	0.750	0.660
abnormal bone density	T1WI	0.785	0.829	0.692	0.834	0.731	0.759
	T2WI	0.866	0.860	0.716	0.861	0.731	0.793
	T1WI+T2WI	0.838	0.842	0.741	0.826	0.752	0.732

## 4 Discussion

In this paper, we develop and test several machine learning models (KNN, SVM, LDA, LR, SGD, Gaussian NB) that could investigate vertebral bone quality based conventional MRI (T1WI, T2WI, T1WI+T2WI) and clinical information (age, BMI) with 160 patients. The research of predicting abnormal bone density and osteoporosis shows that predictive efficacy is sequence-dependent: T1WI features proved most effective for osteoporosis identification, while T2WI features were optimal for abnormal bone density prediction. Notably, combining features from both sequences (T1WI+T2WI) yielded lower AUC values than the best single-sequence models.

The failure of combined T1WI+T2WI to enhance performance contradicts prevailing multi-parametric/sequence imaging trends (4, 25–27), suggesting feature redundancy rather than complementarity (28). Hence, robust prediction models can be feasibly developed using readily available single MRI sequences (T1WI for osteoporosis, T2WI for abnormal bone density), potentially simplifying clinical implementation compared to multi-sequence approaches (29).

This study adopted QCT as the reference standard for bone density assessment, a choice motivated by its inherent advantages over DXA—the conventional gold standard in most related studies. Unlike DXA, which relies on two-dimensional areal BMD measurement, QCT enables three-dimensional volumetric quantification of trabecular bone BMD, free from adjacent tissue interference and thus able to accurately reflect the true bone mass status. A clinical study focusing on elderly males confirmed a markedly higher osteoporosis detection rate with QCT than with DXA(40.3% vs. 13.2%, P<0.001) (30), and another study on postmenopausal women reached the same conclusion(57.9% vs. 50.6%, P = 0.002) (31). In addition, a recent large-scale meta-analysis has further systematically verified the superior sensitivity of QCT over DXA in osteoporosis detection (32). This evidence-based superiority of QCT confers robust anatomical accuracy and high validity on the ground truth of the predictive model.

Bone homeostasis is governed by the dynamic equilibrium between osteoblastic bone formation and osteoclastic resorption (33). Osteoporosis fundamentally disrupts this balance, precipitating accelerated cortical thinning and trabecular microarchitectural deterioration (34, 35). The resultant decline in bone mass manifests as increased cortical porosity and trabecular disconnection, which mechanically compromise skeletal integrity while creating void spaces permissive for bone marrow adipose tissue (BMAT) infiltration (36, 37). Critically, this lipid deposition may establish a self-perpetuating cycle: The infiltration of BMAT can inhibit the differentiation of osteoblasts through changes in regulatory factors in the bone marrow (including cytokines and adipokines) (38), further exacerbate osteoporosis. T1WI’s diagnostic superiority in established osteoporosis likely stems from its dual sensitivity: Short-TE sequences accentuate fat-water contrast, rendering BMAT expansion as hyperintense signals (11, 13, 39); The signal from BMAT enhances the conspicuity of bone trabeculae and microscopic osseous structures (40). The superior spatial resolution of T1-weighted imaging (T1WI) facilitates precise characterization of vertebral texture alterations that directly reflect trabecular microarchitectural deterioration, which surpass the resolution limit of direct visual inspection. This provides a mechanistic basis for T1WI-based osteoporosis prediction: trabecular disintegration and BMAT accumulation generate distinctive textural signatures detectable via radiomics. Notably, this superiority is closely associated with T1WI’s core selected features—Variance-related metrics (quantifying signal heterogeneity) (41). Variance quantifies the dispersion of pixel intensities, reflecting signal variation resulting from patchy fatty infiltration, while Zone Variance measures heterogeneity among contiguous regions of uniform signal, capturing the textural alterations associated with marrow adipose conversion. T1WI’s high sensitivity to fat amplifies the signal contrast between tissues, thereby further enhancing the quantifiability of these Variance-related features. Conversely, T2WI’s efficacy in abnormal bone density detection may operate through edema-sensitive pathways (42). Early bone loss associates with ischemic marrow injury and proinflammatory cytokine surge, these induce vascular hyperpermeability, permitting plasma extravasation that elevates interstitial free water content (43, 44). T2WI’s long-TE weighting amplifies signal in edema-prone regions (42). Consequently, pre-osteoporotic micro-edema generates detectable signal heterogeneity prior to trabecular microarchitectural change or overt fat infiltration. This diagnostic advantage of T2WI its core features—Kurtosis and NonUniformity metrics (Kurtosis: measure of the ‘peakedness’; NonUniformity: texture inhomogeneity) (41). Kurtosis captures scattered local extreme signals induced by microedema against the homogeneous marrow background, whereas NonUniformity metrics quantify the local microscopic texture disorder caused by altered free water content. T2WI’s inherent sensitivity to free water and edema amplifies these subtle signal and texture differences, rendering these features specific quantitative markers for early abnormal bone density.

While X-ray and CT imaging (including low-dose and dual-energy CT) demonstrate outstanding performance in machine learning-based osteoporosis prediction (5, 45–50), their inherent ionizing radiation exposure risks fundamentally constrain widespread clinical adoption, even though excellent results have also been reported using MRI integrated with radiological images (24, 33). In contrast, MRI offers a distinct advantage as a radiation-free alternative, yet research leveraging MRI for vertebral bone quality assessment remains relatively scarce (23, 24). Several pioneering studies have yielded promising results: Zhao et al. employed deep learning-based segmentation on mDixon maps (surpassing traditional T1W and T2WI sequences), achieving impressive AUC-ROCs of 0.925 and 0.899 for predicting abnormal bone density and osteoporosis, respectively (23). Galbusera et al. successfully developed machine learning models for bone disorder/osteoporosis screening using conventional MRI (T1WI + T2WI) alongside radiography, demonstrating excellent predictive power for low bone mineral density (24). Küçükçiloğlu et al. also reported very good predictive outcomes by combining T1WI with CT imaging in deep learning models (33). Notably, Kang et al. conducted a comparative analysis of clinical, radiomic, and combined models, finding that the combined model utilizing T2WI and clinical data achieved the best prediction performance (AUC = 0.913), underscoring the significant potential of T2WI-based machine learning for accurate osteoporosis prediction (22). Collectively, these studies confirm that both T1WI and T2WI MRI sequences are capable of delivering satisfactory predictive performance for osteoporosis and abnormal bone density (18). However, despite these advances, a critical gap remains: the comparative efficacy and potential advantages of sequence-specific predictive models (T1WI vs. T2WI) require dedicated investigation and further exploration.

A recent study investigated the estimation of osteoporosis utilizing deep learning on T1-weighted (T1WI), T2-weighted (T2WI), and Short Time Inversion Recovery (STIR) MRI sequences of the lumbar vertebrae (51). Critically, this research directly compared the predictive performance of these three sequences for osteoporosis diagnosis, reporting T2WI as the optimal sequence. This finding stands in contrast to the results presented in the current article. We speculate that the observed discrepancy may arise from methodological differences between deep learning and machine learning approaches. To be more specific, deep learning approach may capture different texture patterns compared to radiomic feature engineering. Furthermore, the generalizability and persuasiveness of these contrasting findings are potentially limited by the relatively small cohort size (n = 50) in the cited study and its reliance on a single model architecture for comparing T1WI and T2WI performance.

Collectively, these findings support that conventional MRI can enable effective opportunistic screening for osteoporosis. Beyond T1WI and T2WI sequences, recent advancements highlight the broader potential of opportunistic radiomics across imaging modalities. For instance, radiomics data obtained from lumbar MRI ADC maps have demonstrated high efficacy in detecting osteoporosis (52). Similarly, in CT imaging, machine learning models utilizing clivus-radiomic features from routine craniofacial scans have proven highly successful for opportunistic osteoporosis prediction (53).

This study further reveals that both LDA and SGD models exhibited significant discrepancies in performance metrics for detecting abnormal bone density: Despite achieving high AUC values (>0.9), their accuracy was notably lower than that of other models (e.g., KNN, SVM, LR). This phenomenon is primarily attributable to extreme class imbalance (abnormal: normal = 126: 34). Existing literature indicates that certain algorithms fail to accurately capture data distribution characteristics in imbalanced datasets, compromising classification accuracy across categories (53). Specifically, for LDA, the inter-class covariance estimation is dominated by the majority class, which distorts the model’s decision boundary and further leads to frequent misclassification of normal bone density cases, resulting in reduced diagnostic accuracy; for the SGD model, the severe class imbalance disrupts training, as random mini-batch sampling is also heavily biased toward the majority class, resulting in biased feature learning and failure to adequately characterize normal bone density. This directly reduces the diagnostic accuracy and reliability of the SGD model (55–57). These mechanisms collectively degrade model accuracy under imbalance data conditions. In contrast, KNN and SVM maintained robust performance due to their inherent algorithmic advantages: KNN relies on undersampling, which avoids being biased by the data distribution; SVM adopts a kernel-based learning principle, relying on critical boundary samples (support vectors) rather than the entire dataset, thus reducing the impact of the majority class (54, 58). Additionally, LR remained stable through explicit class-weight balancing (class weight = ‘balanced’) in Digital Intelligence Precision Surgery System. All these strategies collectively alleviate the adverse effects of class imbalance, ensuring reliable classification performance.

This study has some limitations need to improve. First, as previously demonstrated, data imbalance adversely affected LDA and SGD performance. Future prospective studies with a more balanced sample size are needed to address this limitation and optimize model performance. Second, the dataset employed for classification models is relatively small, potentially affecting the generalizability of the models. Third, the lack of external validation data further undermines the reliability of the results. Future work should enroll larger sample sizes and perform external validation to enhance osteoporosis prediction accuracy and sensitivity.

## 5 Conclusion

This preliminary study indicates that conventional lumbar MRI sequences could have sequence−dependent diagnostic value: T1WI exhibits better performance in identifying advanced bone loss, while T2WI is more effective for abnormal bone density prediction. Our sequence-specific approach suggests the feasibility of opportunistic osteoporosis screening during routine lumbar MRI, avoiding additional radiation exposure from QCT/DXA.

## Data Availability

The original contributions presented in the study are included in the article/**Supplementary Material**. Further inquiries can be directed to the corresponding author/s.
