# Supplementary material for: Sequence-specific radiomics for diagnosis of spinal bone loss
Source: Front Endocrinol (Lausanne). 2026 Apr 29;17:1823826. doi: 10.3389/fendo.2026.1823826 (PMC13167201; doi:10.3389/fendo.2026.1823826)
Supplement: Supplementary file 1 [file DataSheet1.pdf]

Supplementary Material

Sequence-Specific Radiomics for diagnosis of Spinal Bone Loss

Tingyu Xue[1]†, Yaguang Li[1]†, Huayi Zhao[1], Tianzi Zhang[1], Jiayi Wang[1], WenHao Jiang[1], Licun Lei[1,2]*, Yong Wang[1,2]*

[1]Department of Radiology and Nuclear Medicine, The First Hospital of Hebei Medical University, Hebei 050000, China

[2]School of Medical Imaging, Hebei Medical University, Hebei 050017, China

†Tingyu Xue and Yaguang Li contributed equally to this work.

*** Correspondence:**
Licun Lei
57900264@hebmu.edu.cn

Yong Wang
wangyong@hebmu.edu.cn

1 Supplementary Figures and Tables

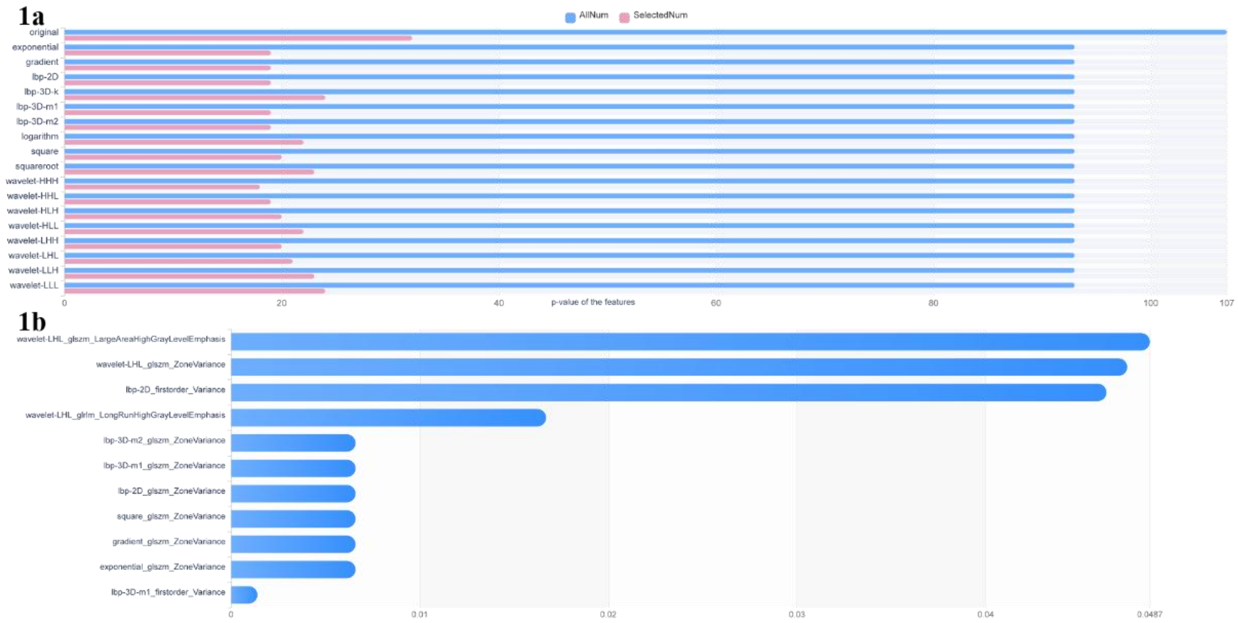

Fig. S1 VarianceThreshold (a) and SelectKBest (b) althorithm in 10-fold cross-validation classification model for osteoporosis with T1WI.

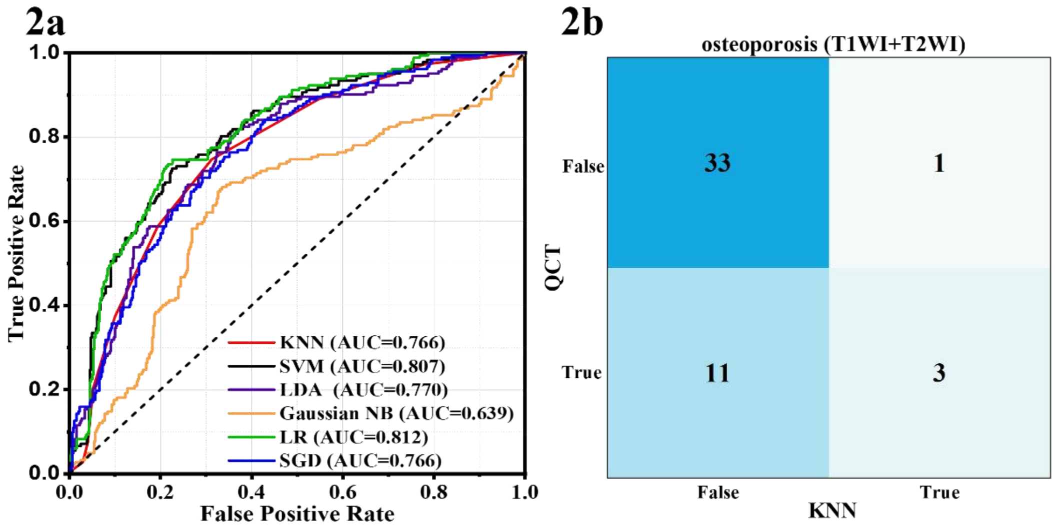

Fig. S2 Receiver operating characteristic curves (a) and confusion matrices (b) of the six machine learning models for the prediction of osteoporosis based on clinical data and T1WI + T2WI.

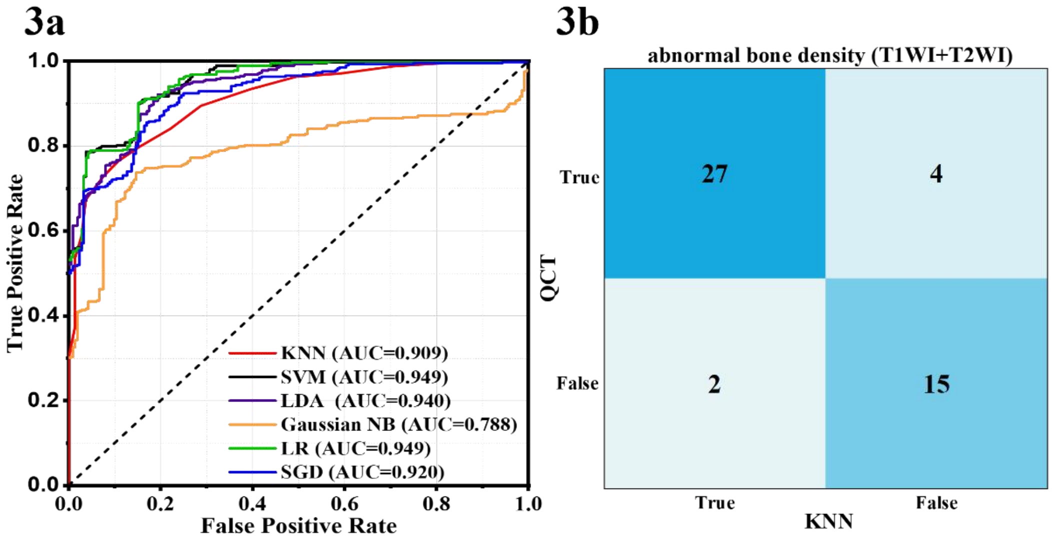

Fig. S3 Receiver operating characteristic curves (a) and confusion matrices (b) of the six machine learning models for the prediction of abnormal bone density based on clinical data and T1WI + T2WI.

Table S1 Mean performance metrics for osteoporosis prediction on test set (10 repeated evaluations).

		KNN	SVM	LDA	LR	SGD	Gaussian NB
T1WI	AUC-ROC	0.821	0.834	0.836	0.835	0.820	0.821
	specificity	0.862	0.743	0.796	0.773	0.839	0.905
	sensitivity	0.555	0.810	0.722	0.764	0.546	0.398
	accuracy	0.761	0.768	0.771	0.761	0.761	0.733
	F1-score	0.607	0.697	0.679	0.691	0.573	0.497
T2WI	AUC-ROC	0.782	0.811	0.808	0.817	0.808	0.752
	specificity	0.947	0.704	0.842	0.782	0.858	0.892
	sensitivity	0.270	0.828	0.519	0.711	0.445	0.354
	accuracy	0.724	0.744	0.733	0.759	0.721	0.715
	F1-score	0.391	0.676	0.559	0.656	0.505	0.430
T1WI+ T2WI	AUC-ROC	0.775	0.819	0.781	0.816	0.780	0.700
	specificity	0.938	0.738	0.797	0.918	0.813	0.825
	sensitivity	0.296	0.762	0.629	0.467	0.594	0.258
	accuracy	0.748	0.745	0.748	0.751	0.750	0.660
	F1-score	0.403	0.636	0.595	0.562	0.581	0.253

Table S2 Mean performance metrics for abnormal bone mass prediction on test set (10 repeated evaluations).

		KNN	SVM	LDA	LR	SGD	Gaussian NB
T1WI	AUC-ROC	0.884	0.929	0.910	0.938	0.895	0.881
	specificity	0.818	0.852	0.985	0.889	0.986	0.779
	sensitivity	0.772	0.818	0.563	0.808	0.618	0.804
	accuracy	0.785	0.829	0.692	0.834	0.731	0.759
	F1-score	0.834	0.869	0.717	0.871	0.761	0.846
T2WI	AUC-ROC	0.942	0.955	0.963	0.962	0.937	0.932
	specificity	0.874	0.888	0.992	0.894	0.974	0.914
	sensitivity	0.861	0.848	0.574	0.843	0.590	0.705
	accuracy	0.866	0.860	0.716	0.861	0.731	0.793
	F1-score	0.897	0.892	0.726	0.891	0.735	0.801
T1WI+ T2WI	AUC-ROC	0.923	0.948	0.942	0.949	0.925	0.837
	specificity	0.847	0.857	0.971	0.904	0.969	0.564
	sensitivity	0.834	0.832	0.623	0.785	0.642	0.816
	accuracy	0.838	0.842	0.741	0.826	0.752	0.732
	F1-score	0.874	0.872	0.758	0.856	0.772	0.803

Table S3 The selected features are used in the prediction of osteoporosis.

T1WI	T2WI	T1WI+T2WI
lbp-3D-m1_firstorder_Variance-10*	wavelet-LLH_firstorder_Kurtosis-10	square_firstorder_TotalEnergy-9
lbp-2D_firstorder_Variance-2 exponential_glszm_ZoneVariance-10	wavelet-HHH_glszm_LargeAreaLowGrayLevelEmphasis-9	square_firstorder_Kurtosis-8
LHL_glszm_LargeAreaHighGrayLevelEmphasis-4	lbp-3D-k_firstorder_Range-6	square_firstorder_Skewness-8
LHL_glrlm_LongRunHighGrayLevelEmphasis-4	wavelet-LHH_firstorder_Kurtosis-6	wavelet-HLH_firstorder_Kurtosis-8
gradient_glszm_ZoneVariance-6	wavelet-LLH_glszm_SizeZoneNonUniformity-6	exponential_firstorder_Kurtosis-7
square_glszm_ZoneVariance-6	wavelet-HLL_glszm_SizeZoneNonUniformity-5	original_firstorder_Kurtosis-7
lbp-3D-m2_firstorder_Variance-2	exponential_glszm_GrayLevelNonUniformity-4	square_firstorder_Energy-7
lbp-2D_glszm_ZoneVariance-3	original_shape_Maximum2DDiameterRow-4	gradient_firstorder_Kurtosis-5
lbp-3D-m1_glszm_ZoneVariance-3	wavelet-HLH_gldm_LargeDependenceHighGrayLevelEmphasis-4	wavelet-LLH_glrlm_LongRunHighGrayLevelEmphasis-5
lbp-3D-m2_glszm_ZoneVariance-2	lbp-3D-m2_firstorder_Variance-4	original_firstorder_TotalEnergy-3
LHL_gldm LargeDependenceHighGrayLevelEmphasis-2	wavelet-LLH_gldm_LargeDependenceEmphasis-4	wavelet-LHL_firstorder_TotalEnergy-3
wavelet-LHL_glrlm LongRunEmphasis-1	exponential_glszm_LargeAreaEmphasis-3	wavelet-LLL_firstorder_TotalEnergy-3
wavelet-LLH firstorder Energy-2	lbp-3D-k_firstorder_Maximum-3	wavelet-LLH_gldm_LargeDependenceHighGrayLevelEmphasis-2
	original_firstorder_Kurtosis-3	wavelet-HHH_glszm_LargeAreaEmphasis-2
	wavelet-LLL_gldm_LargeDependenceEmphasis-3	wavelet-HHH_glszm_LargeAreaHighGrayLevelEmphasis-2
	exponential_glszm_LargeAreaLowGrayLevelEmphasis-2	wavelet-HHH_glszm_LargeAreaLowGrayLevelEmphasis-2
	gradient_glszm_GrayLevelNonUniformity-2	wavelet-HHL_glszm_LargeAreaEmphasis-2
	lbp-3D-k_gldm_DependenceVariance-2	wavelet-HHL_glszm_LargeAreaHighGrayLevelEmphasis-2
	logarithm_firstorder_TotalEnergy-2	wavelet-HHL_glszm_LargeAreaLowGrayLevelEmphasis-2
	original_shape_SurfaceArea-2	wavelet-HLH_glszm_SizeZoneNonUniformity-2
	square_glszm_GrayLevelNonUniformity-2	wavelet-LLH_glrlm_RunVariance-2
	wavelet-HLH_gldm_LargeDependenceLowGrayLevelEmphasis-2	original_shape_MajorAxisLength-1
	wavelet-HLL_glszm_SizeZoneNonUniformity-2	wavelet-LLH_gldm_LargeDependenceLowGrayLevelEmphasis-1
	wavelet-LLL_firstorder_Kurtosis-2	wavelet-LLL_ngtdm_Busyness-1
	exponential_glszm_LargeAreaEmphasis-2	lbp-3D-k_gldm_DependenceVarianc-1
	gradient_glszm_LargeAreaEmphasis-1	lbp-3D-m1_firstorder_Kurtosis 1
	gradient_glszm_LargeAreaHighGrayLevelEmphasis-1	logarithm_glszm_LargeAreaLowGrayLevelEmphasis-1
	lbp-2D_glszm_GrayLevelNonUniformity-1	logarithm_glszm_SizeZoneNonUniformity-1
	lbp-2D_glszm_LargeAreaEmphasis-1	original_glszm_LargeAreaLowGrayLevelEmphasis-1
	lbp-2D_glszm_LargeAreaHighGrayLevelEmphasis-1	original_glszm_SizeZoneNonUniformity-1
	lbp-3D-m1_firstorder_Variance-1	squareroot_glszm_LargeAreaLowGrayLevelEmphasis-1
	lbp-3D-m1_glszm_GrayLevelNonUniformity-1	squareroot_glszm_SizeZoneNonUniformity-1
	lbp-3D-m2_glszm_GrayLevelNonUniformity-1	wavelet-HHL_firstorder_Kurtosis-1
	lbp-3D-m2_firstorder_Variance-1	wavelet-LLH_glrlm_LongRunEmphasis-1
	logarithm_firstorder_TotalEnergy-1	wavelet-LLH_glszm_SizeZoneNonUniformity-1
	original_shape_Maximum2DDiameterColumn-1	wavelet-LLL_glszm_LargeAreaEmphasis-1
	square_glszm_LargeAreaLowGrayLevelEmphasis-1	wavelet-LLL_glszm_LargeAreaLowGrayLevelEmphasis-1
	wavelet-HHL_glszm_LargeAreaLowGrayLevelEmphasis-1	
	wavelet-HLH_gldm_LargeDependenceEmphasis-1	
	wavelet-HLH_glszm_LargeAreaHighGrayLevelEmphasis-1	
	wavelet-HLH_glszm_SizeZoneNonUniformity-1	
	wavelet-HLL_gldm_LargeDependenceHighGrayLevelEmphasis-1	

*-10 means feature occurs ten times.

Table S4 The selected features are used in the prediction of abnormal bone mass.

T1WI	T2WI	T1WI+T2WI
original_shape_Maximum2DDiameterColumn-8	exponential_firstorder_Kurtosis-7	original_shape_Maximum3DDiameter-6
original_shape_MinorAxisLength-8	wavelet-LLL_glszm_SizeZoneNonUniformity-6	lbp-2D_firstorder_Variance-6
squareroot_firstorder_Range-8	exponential_glszm_GrayLevelNonUniformity-4	exponential_firstorder_TotalEnergy-6
gradient_firstorder_Kurtosis-7	original_shape_MinorAxisLength-3	original_firstorder_TotalEnergy-5
wavelet-LLL_gldm_LargeDependenceEmphasis-7	original_firstorder_Kurtosis-3	wavelet-LLL_firstorder_TotalEnergy-5
lbp-3D-m1_firstorder_Variance-6	wavelet-HLH_gldm_LargeDependenceHighGrayLevelEmphasis-1	bp-3D-k_firstorder_TotalEnergy-5
wavelet-LLH_firstorder_Range-6	wavelet-LLL_firstorder_Kurtosis-1	exponential_firstorder_TotalEnergy-5
wavelet-LLL_gldm_LargeDependenceHighGrayLevelEmphasis-5	wavelet-HLL_firstorder_Range-1	logarithm_firstorder_TotalEnergy-4
wavelet-LLH_firstorder_Minimum-5	square_firstorder_Kurtosis-1	squareroot_firstorder_TotalEnergy-4
wavelet-HHH_glszm_GrayLevelNonUniformity-3	wavelet-LHL_firstorder_Energy-1	wavelet-LLH_glszm_SizeZoneNonUniformity-4
wavelet-LLH_gldm_LargeDependenceHighGrayLevelEmphasis-3	gradient_glszm_GrayLevelNonUniformity-1	logarithm_firstorder_TotalEnergy-3
original_firstorder_Kurtosis-2	square_glszm_GrayLevelNonUniformity-1	wavelet-LLL_firstorder_90Percentile-3
wavelet-HLH_gldm_DependenceVariance-2	lbp-2D_glszm_GrayLevelNonUniformity-1	original_shape_Maximum2DDiameterSlice-2
wavelet-LLH_firstorder_TotalEnergy-2	lbp-3D-m1_glszm_GrayLevelNonUniformity-1	original_shape_Maximum2DDiameterRow-2
lbp-3D-k_gldm_LargeDependenceLowGrayLevelEmphasis-1	wavelet-HLH_glszm_GrayLevelNonUniformity-1	wavelet-LLL_glszm_GrayLevelNonUniformity-2
lbp-3D-m2_firstorder_Variance-1	wavelet-HHH_glszm_SizeZoneNonUniformity-1	squareroot_firstorder_TotalEnergy-2
wavelet-HHH_gldm_LargeDependenceHighGrayLevelEmphasis-1	original_shape_MajorAxisLength-1	squareroot_glszm_GrayLevelNonUniformity-1
wavelet-HHL_gldm_LargeDependenceHighGrayLevelEmphasis-1	lbp-3D-m2_glszm_GrayLevelNonUniformity-1	logarithm_glszm_GrayLevelNonUniformity-1
wavelet-LHL_gldm_LargeDependenceHighGrayLevelEmphasis-1		original_glszm_GrayLevelNonUniformity-1
lbp-3D-k_gldm_DependenceVariance-1		wavelet-LLH_glszm_GrayLevelNonUniformity-1
wavelet-LHH_firstorder_Energy-1		wavelet-HHL_glszm_GrayLevelNonUniformity-1
wavelet-LHH_firstorder_TotalEnergy-1		wavelet-HHH_glszm_GrayLevelNonUniformity-1
wavelet-HLL_firstorder_TotalEnergy-1		original_shape_SurfaceArea-1
wavelet-HLL_glszm_GrayLevelNonUniformity-1		original_shape_Maximum2DDiameterSlice-1
lbp-3D-m1_firstorder_InterquartileRange-1		
wavelet-LLH_firstorder_InterquartileRange-1		
